# Microglia in Glioblastomas: Molecular Insight and Immunotherapeutic Potential

**DOI:** 10.3390/cancers16111972

**Published:** 2024-05-22

**Authors:** Sabrina Nusraty, Ujwal Boddeti, Kareem A. Zaghloul, Desmond A. Brown

**Affiliations:** Surgical Neurology Branch, National Institute of Neurological Disorders and Stroke, National Institutes of Health, Bethesda, MD 20892, USA; sabrina.nusraty@nih.gov (S.N.); ujwalboddeti@som.umaryland.edu (U.B.); kareem.zaghloul@nih.gov (K.A.Z.)

**Keywords:** glioblastoma, immunotherapy, microglia, tumor-associated microglia

## Abstract

**Simple Summary:**

Glioblastoma (GBM) is the most aggressive primary brain tumor. Despite intensive management with surgery, radio-, and chemotherapy, prognosis remains bleak, with a median survival of 15 months. Recently, microglia, the resident immune cells of the brain, have emerged as a potential therapeutic target for treatment of GBM. However, precisely how microglia interact with GBM cells and may be exploited for novel immunotherapies is not well understood. Here, we discuss the role of microglia in the GBM tumor microenvironment (TME), focusing particularly on the pro- and anti-tumorigenic pathways involved. We first introduce microglia and their role in the normal brain environment. Next, we discuss the microglia-GBM crosstalk, delving into specific factors that mediate these interactions. Finally, we present a comprehensive discussion of suitable microglial pathways that can be targeted to modulate the immune response in the TME to halt tumor progression. We discuss this in the context of current and past clinical trials targeting microglia, summarizing both successes and failures, and highlight promising pathways that are yet to be explored, suggesting future directions for study.

**Abstract:**

Glioblastoma (GBM) is one of the most aggressive and devastating primary brain tumors, with a median survival of 15 months following diagnosis. Despite the intense treatment regimen which routinely includes maximal safe neurosurgical resection followed by adjuvant radio- and chemotherapy, the disease remains uniformly fatal. The poor prognosis associated with GBM is multifactorial owing to factors such as increased proliferation, angiogenesis, and metabolic switching to glycolytic pathways. Critically, GBM-mediated local and systemic immunosuppression result in inadequate immune surveillance and ultimately, tumor-immune escape. Microglia—the resident macrophages of the central nervous system (CNS)—play crucial roles in mediating the local immune response in the brain. Depending on the specific pathological cues, microglia are activated into either a pro-inflammatory, neurotoxic phenotype, known as M1, or an anti-inflammatory, regenerative phenotype, known as M2. In either case, microglia secrete corresponding pro- or anti-inflammatory cytokines and chemokines that either promote or hinder tumor growth. Herein, we review the interplay between GBM cells and resident microglia with a focus on contemporary studies highlighting the effect of GBM on the subtypes of microglia expressed, the associated cytokines/chemokines secreted, and ultimately, their impact on tumor pathogenesis. Finally, we explore how understanding the intricacies of the tumor-immune landscape can inform novel immunotherapeutic strategies against this devastating disease.

## 1. Introduction

Glioblastoma (GBM) is the most aggressive malignant primary brain tumor, with a median survival of 15 months following diagnosis [1,2]. Classified by the World Health Organization (WHO) as a grade IV astrocytoma, GBM is histologically characterized by marked microvascular proliferation and necrosis [1]. Given its highly infiltrative nature, GBM often portends very poor clinical outcomes, carrying a five-year survival rate of less than 5% [3]. Management of newly diagnosed GBM consists of surgical resection, if possible, followed by a combination of radio- and chemotherapy [1,4]. The most recent significant change to standard of care was driven by a landmark clinical trial in 2005, which demonstrated that concomitant temozolomide (TMZ) treatment conferred a survival benefit to GBM patients [4]. However, despite aggressive interventions, outcomes have remained largely stagnant, with rates of survival beyond five years showing no apparent improvement. Given this, it is imperative that we better understand the pathomechanisms underlying GBM, with the aim of driving novel therapeutic interventions. More recently, efforts have begun looking at the immunological response to see if the unique recruitment of immune cells to the tumoral and peritumoral environments can be used to slow or halt tumor progression, bringing us to the resident immune cells of the brain—microglia.

In the central nervous system (CNS), there are two main types of macrophages, namely microglia and CNS-associated macrophages (CAMs). Microglia are parenchymal macrophages that originate from myeloid precursors, whereas CAMs are found outside the parenchyma at CNS interfaces. Of the two, microglia are the most well-studied, due in part to single-cell omics studies which have shed light on their specific functions and phenotypes, as we discuss below. Microglia are the macrophages of the central nervous system (CNS) that serve to monitor and interact with neurons, keeping tissue metabolism, inflammation, and cell death in the CNS in check [5]. Although macrophages are intended to coordinate the host’s immune defense, they often become irregularly activated in the context of cancer and have been shown to contribute to immune dysregulation and the progression of disease [6]. In fact, higher levels of tumor-associated macrophages (TAMs) have been reported to be an independent prognostic factor in several types of cancers [7]. Recently, studies have investigated the factors secreted by the tumor microenvironment in GBM that directly impact the function of microglia. Through in vivo murine models and in vitro human experimentation, Dumas et al. demonstrated that glioma initiating cells stimulated the mTOR signaling pathway in microglia, contributing to tumor immune evasion, reduced T-cell proliferation, and impaired immune reactivity [7]. Additionally, glial cell line-derived neurotrophic factor (GDNF), a factor highly expressed in human gliomas, was found to (i) recruit microglial cells to the tumoral site, (ii) significantly increase tumor growth, and (iii) decrease survival in murine models [8]. By understanding the factors through which tumor cells communicate with microglia to evade immune surveillance, researchers can identify therapeutic targets that have the potential to reduce the rapid proliferation and spread that is associated with GBM.

Over recent years, the neuroinflammatory profile of GBM has garnered much scientific interest. Specifically, recent studies have identified numerous signaling pathways that are commonly implicated in GBM to be strongly correlated with the immune functions of microglia. As such, targeting microglia-associated pathways is coming into the limelight as a promising approach to alleviate disease progression and hinder tumor burden. In this review, we explore the current state of knowledge regarding the tumor-suppressive and -promoting functions of microglia in the context of GBM pathogenesis. Namely, we discuss the vastly different M1 and M2 phenotypes and the role each play in the context of glioma progression. Finally, we conclude by discussing potential targets for intervention and discuss future therapeutic efforts that utilize and/or target microglia in patients with GBM.

## 2. Role of Microglia in Normal Brain Function

**Figure 1:**
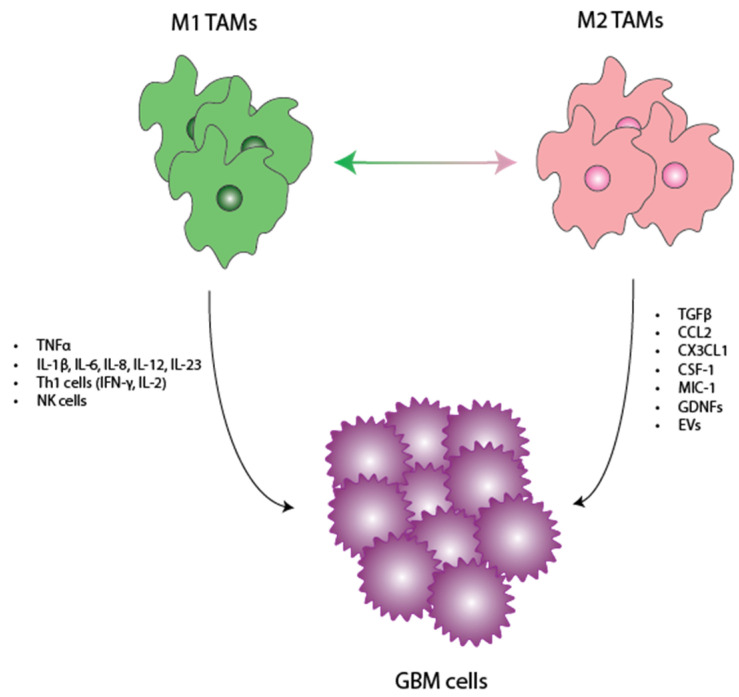
Interplay between TAMs and GBM. Here, we show a schematic delineating the factors/cytokines released by M1 and M2 TAMS. We discuss how these individual factors functionally exert antitumoral and pro-tumoral effects in the TME. TAMs—tumor-associated macrophages, GBM—glioblastoma, TME—tumor microenvironment.

Microglia are known to be the resident immune cells of the CNS. They have a host of functions that regulate brain development as they engage in dynamic interactions with neurons and glia, maintain neural networks, and coordinate injury-repair responses [9]. In the developing brain, microglia actively survey and prune excess synapses which optimizes neural circuits and allows for proper wiring of the nervous system. Microglia play an essential role in modulating synaptic transmission by detecting changes in neural activity and responding through the release of cytokines and chemokines which can alter the strength of connections among neighboring neurons. Recent studies have also found significant microglial involvement in processes such as neuro- and oligodendrogenesis, remyelination, and angiogenesis [10,11]. In conditions of neuronal injury, it has been well established that purinergic signaling between neurons and microglia significantly influence the injury-response process [12]. Specifically, ATP and its metabolites that are released from compromised neurons interact with P2X receptors and P2Y G-protein coupled receptors (GPCRs) on microglia, ultimately influencing microglial transition from the quiescent to active state [13,14]. However, in addition to mediating key physiologic changes in the CNS, microglia are also largely implicated in pathological states of neurological disease, such as epilepsy, Alzheimer’s Disease (AD), and brain tumors, as we discuss later. In addition to maintaining the brain’s microenvironment, microglia are essential in coordinating the immune response of the CNS through their phagocytic and signaling capabilities [15,16]. The phagocytic receptors expressed by microglia prompt their migration toward signals released by apoptotic cells, myelin debris, damaged neurites, pathogens, and other damage-associated indicators [17]. Through a process of recognition, engulfment, digestion, and response, microglia clear unwanted signals and maintain a healthy environment within the CNS [18]. While microglia are continuously working to optimize the health of the CNS, their activities can be distinctly categorized into inactive and active phenotypes [19]. When activated, microglia develop rounded cell bodies and retract their processes, develop phagocytic abilities, and upregulate the expression of major histocompatibility complex (MHC) class 1 and 2 antigens to communicate with T-cells [20]. In the inactive state, microglia continuously survey their environment, extending and retracting their long, thin processes to monitor their surroundings [21]. Depending on the aberrant signal detected, microglia undergo morphological changes and release immune mediators as they transition into the active state, which entails microglia assuming one of a range of phenotypes, including classically activated M1 (pro-inflammatory) or alternatively activated M2 (anti-inflammatory) [21]. M1 microglia release pro-inflammatory cytokines (interleukins 1β (IL-1β) and 6 (IL-6), tumor necrosis factor-alpha (TNF-α)) and chemokines (macrophage inflammatory protein (MIP)-1α, monocyte chemoattractant protein (MCP)-1) to activate repair mechanisms and promote neuronal death [22]. Conversely, M2 microglia engage in a neuroprotective and regenerative role, being associated with increased levels of anti-inflammatory cytokines (IL-4, IL-13, GDNF, insulin-like growth factor-1 (IGF-1)) and neurotrophic factors (Arg-1, Ym-1, CD200R, and IL-10) to promote growth and healing [23]. While M1 and M2 microglia result in different effects on the brain’s microenvironment, recent studies are beginning to suggest that these are not two distinct phenotypes, but rather mark opposite ends of a spectrum along which a wide variety of intermediate microglia subtypes exist (Figure 1) [24]. Furthermore, the dynamic transition from M1 to M2 microglial phenotypes has garnered significant attention in the context of better understanding inflammatory responses in neurological disorders such as Alzheimer’s disease (AD), Parkinson’s disease (PD), epilepsy, and GBM, with the particular microglial phenotypes recruited depending specifically on the disease state in question. Below, we discuss the specific microglial makeup in the context of the GBM microenvironment.

## 3. Role of Microglia in Glioma Progression

As discussed above, TAMs are canonically categorized into two main phenotypes—pro-inflammatory M1 and anti-inflammatory M2. TAMs are typically studied and characterized by their presence in the necrotic tumoral core; however, they are also found in the surrounding TME, including near the tumor microvasculature and periphery [25]. Typically, TAMs in the tumoral core are macrophages with a M2-like phenotype, whereas TAMs near the tumor periphery are mainly microglia with a M1-like phenotype [26,27]. However, more importantly, what sets these two subgroups apart in the context of glioma progression is their respective roles in tumor inhibition and proliferation, as we will discuss below.

### 3.1. Tumor-Promoting Role of Microglia

Traditionally, M2 TAMs have been understood to play pro-tumorigenic functions by secreting anti-inflammatory factors, such as TGF-β, which regulate the responses of regulatory T-cells (Tregs) and myeloid-derived suppressor cells (MDSCs) [28]. More importantly, these factors also work to inhibit CD8+ cytotoxic T-cells. Although lymphoid cells comprise less than 2% of the total tumor, this subpopulation is largely made up of CD8+ cytotoxic T-cells and Tregs. Although CD8+ cytotoxic T-cells normally function in eradicating abnormal cells, such as cancer cells, in the context of GBM, these cells are largely dysfunctional. This dysfunction is further worsened with M2 TAM-mediated inhibition, further diminishing CD8+ cytotoxic T-cells’ role, allowing aberrant tumor cells to continue to proliferate unchecked [29,30]. An additional critical role of TGF-β is the function it plays in promoting tumor invasion by upregulating matrix metalloprotein (MMP)-2, a protein shown to play important roles in neo-angiogenesis and tumor vascularization [31]. In fact, MMP2 upregulation has been shown to be strongly associated with tumor malignancy and metastasis [32,33]. However, perhaps the strongest evidence of the tumor-promoting role of M2 TAMs is seen in their impacts on patient survival.

Recent studies have shown that greater infiltration of M2 TAMs is associated with significantly inferior survival in glioma patients [34,35]. Furthermore, when tissue samples from patients with gliomas were considered, those from patients with higher grade gliomas stained for significantly higher amounts of M2-specific markers, such as CD204 [36]. These findings are not only limited to gliomas but are also seen across cancers as well. For example, the M2-specific CD204 marker’s presence was associated with poor prognosis in patients with pancreatic adenocarcinoma, hepatocellular carcinoma, and lung adenocarcinoma, suggesting that the pro-tumorigenic role of M2 TAMs is not only restricted to gliomas but extends extracranially as well [37]. However, to better understand the specific mechanisms by which M2 TAMs promote tumor growth, it is important to understand how they communicate with GBM cells.

Given that M2 TAMs greatly support and promote glioma progression, GBM cells have mechanisms to recruit and polarize them. This is accomplished through the release of the following: (1) C-C motif chemokine ligand 2 (CCL2), (2) C-X3-C motif chemokine ligand 1 (CX3CL1), (3) colony-stimulating factor 1 and 2 (CSF1,2), (4) macrophage inhibitory cytokine-1 (MIC-1), (5) GDNF, and (6) extracellular vesicles (EVs). We will discuss each in turn below.

#### 3.1.1. CCL2 and CX3CL1

Studies have shown that of the cytokines released by GBM cells, the most important chemoattractants are CCL2 and CX3CL1 [38]. CCL2 works by binding to the C-C motif chemokine receptor 2 (CCR2), activating a number of downstream pathways that ultimately results in supporting migration and recruitment of monocytes to the TME [39]. In addition to playing a role in direct TAM recruitment, CCL2 also induces microglial release of IL-6, which in turn increases glioma cell invasiveness [40].

Similar to CCL2, CX3CL1 works by binding to CX3C receptor 1 (CX3CR1) on TAMs to recruit them to the TME. More specifically, studies have shown that the CX3CL1/CX3CR1 axis is overexpressed in invading glioma cells, and when treated with neutralizing antibodies, this invasiveness is largely reduced [41]. More directly, in vivo studies have shown that co-expression of CX3CL1 and CX3CR1 led to a more malignant tumor phenotype with increased microglia/macrophage infiltration and microvessel density, and shorter overall survival [42]. However, recent studies have also suggested that the role of CX3CL1 in glioma progression may be more complicated, with Sciume et al. showing that TGF-β may exert its pro-tumorigenic effects by directly counteracting CX3CL1 activity, resulting in increased tumor invasion, suggesting that the CX3CL1/CX3CR1 axis may also play an inhibitory role in tumor progression [41].

#### 3.1.2. CSF1 and CSF2

CSF1 has been shown to be produced directly by glioma cells and works by binding to the CSF1 receptor (CSF1R) to regulate monocyte and microglial migration and promote TAM polarization towards the M2 phenotype [43,44]. In fact, Komohara et al. recently showed that CSF1 expression levels were in fact correlated with glioma grade, with higher grade corresponding to higher CSF1 expression [44]. In addition, CSF1 has been shown to not only promote TAM polarization towards the M2 phenotype but also induce cell survival and tumor proliferation via an autocrine mechanism, likely further explaining its correlation with high tumor grade [44]. Similarly, CSF2 has also been found to be overexpressed in GBM, with it also being associated with inducing pro-tumorigenic polarization of macrophages [44].

#### 3.1.3. MIC-1

MIC-1 is a member of the TGF-β family and is associated with microglial recruitment, inhibition of their phagocytic activities, and promotion of TGF-β secretion, ultimately favoring an immunosuppressive phenotype [45]. Additionally, MIC-1 RNA expression was found to progressively increase throughout malignant glioma progression [46,47].

#### 3.1.4. GDNF

GDNF is secreted by glioma cells and has been shown to attract microglia to the TME by acting on GDNF family receptor alpha 1 and 2. In fact, in vitro studies by Ku et al. showed that glioma cells with downregulated GDNF expression, when implanted into mouse brains, resulted in significantly reduced microglial recruitment, ultimately translating to improved survival, suggesting the critical role GDNF plays and the therapeutic potential of targeting this pathway [8].

#### 3.1.5. Extracellular Vesicles (EVs)

EVs are particles that are largely composed of exosomes derived from endosome, ectosome, or microvesicle origin. Of the different types, “small EVs” (sEVs) are the most abundant and biologically relevant. EVs are canonically known to carry lipids, proteins, nucleic acids, and metabolites. They serve as mediators of cell–cell communication, with studies suggesting that sEVs play key roles in mediating physiologic and pathologic processes by affecting immune responses, neuronal communication, and regulation, among other functions. In the context of GBM, in addition to recruitment of TAMs to the TME via the release of secretory proteins, as discussed above, GBM-derived stem cells (GSCs) have been shown to release sEVs into the TME to polarize TAMs to the pro-tumorigenic M2-phenotype [48]. These are specifically referred to as tumor-derived sEVs (TDsEVs). After release, TDsEVs are phagocytosed by monocytes, where they have a number of downstream effects. For example, PD-L1 expressed on TDsEVs has been shown to actively inhibit activation and proliferation of CD8+ cytotoxic T-cells, further blunting the anti-tumor immune response [49]. Additional studies have shown that GSC-derived TDsEVs also carry microRNAs (miRNAs), non-coding RNAs that are involved in regulating gene expression. Specifically, studies have identified specific miRNAs (i.e., miR-21, miR-451, and miR-1246) implicated in polarizing monocytes to the M2-phenotype [49,50]. In addition to tumor cell release of sEVs, immune cells also release sEVs in the tumor-immune cell crosstalk. Studies have shown that M2 TAMs release sEVs (M2sEVs) which can also polarize macrophages towards low-IL-12, high-IL-10 phenotypes, causing release of Th2 cytokines. Furthermore, M2sEVs have been shown to effectively promote tumoral migration. Overall, TDsEVs and M2sEVs provide key examples of the role of EVs in modulating the immune response to facilitate GBM tumor growth.

As discussed here, GBM cells have a myriad of mechanisms through which they recruit and polarize TAMs to promote tumoral growth and proliferation. However, contrary to M2 TAMs, M1 TAMs have been shown to play a vastly different role in the context of glioma progression, as we will discuss below.

### 3.2. Tumor-Inhibiting Role of Microglia

When discussing TAMs, the literature largely discusses their role in tumor progression; however, there is another functional side to these diverse players in the TME. In contrast to TAMs exhibiting an M2, pro-tumoral phenotype, M1 TAMs are classically associated with playing an anti-tumor role by secreting factors known to inhibit tumor growth, such as TNF-α, IL-1β, IL-6, IL-8, IL-12, and IL-23 [51]. They are found in abundant quantities in early stages of tumor development, largely being overtaken by M2 TAMs in later stages [52]. Furthermore, through the secretion of pro-inflammatory cytokines, M1 TAMs have been shown to promote T helper 1 (Th1) and natural killer (NK) cell responses [49]. Th1 cells are known for their role in inducing cellular immunity by producing IFN-γ and IL-2 [53]. Notably, high Th1 cell populations have been shown to delay tumor progression, with a reduced Th1/Th2 ratio being associated with a poor prognosis in patients with GBM [54].

In addition to promoting the Th1 response, M1 TAMs also potentiate the NK response, which is well known to play a strong anti-tumor role, especially in the context of GBM. This is largely due to their ability to overcome the immunosuppressive environment of the GBM TME. For starters, NK cells are uniquely poised to recognize tumor cells without antigen sensitization based on the balance of what are referred to as activating (i.e., natural cytotoxicity receptors) and inhibitory receptors [55]. As such, NK cells can still function in the setting of diverse intratumoral heterogeneity. Further, after detection, NK cell-mediated killing is highly efficient, with a single released granule from an activated NK cell being capable of killing a single target tumor cell [56]. In fact, because of these advantages, several clinical trials have investigated NK-cell based immunotherapy in the management of GBM.

Similar to M2 TAMs, M1 TAMs have also been shown to release sEVs (M1sEVs). Specifically, M1sEVs have been shown to promote T-cell proliferation, generation of memory T-cells, and polarization of CD4+ T-cells towards the Th17 phenotype. Furthermore, M1sEVs have been shown to increase expression of pro-inflammatory cytokines (ex. IL-6, IL-12, and INF-γ) and decrease expression of IL-4 and IL-10. Finally, M1sEVs have been shown to polarize macrophages to the M1 phenotype and significantly inhibit tumor growth. Overall, these are just some of the many mechanisms by which M1 TAMs exhibit an anti-tumor role in the TME.

## 4. Targeting Microglia in Novel Immunotherapeutics for Glioblastoma

One of the main challenges in treating GBM is its marked molecular heterogeneity, making it particularly difficult to treat, resulting in high rates of post-treatment recurrence. However, TAMs, which make up a large proportion of GBM tumors (30–50%), are by contrast genetically stable, making them relatively simpler to target [57,58]. Given this and the many molecular pathways available to target, there has been a recent shift in GBM research, as reflected by the explosion of recent preclinical and clinical studies exploring targeting TAMs (Section 3). Here, we summarize the current clinical trials aimed at targeting TAMs in GBM, discussing TAM-related pathways that have been considered. Then, we conclude by suggesting future directions, with promising TAM targets that are yet to be explored (Table 1).

To-date, there have been a handful of studies conducted exploring the potential benefit of TAM-pathway targeting in achieving improved tumor outcomes. However, many of the therapeutics explored in these studies have shown marginal, if any, benefit in human trials. For example, tetracycline has been shown to inhibit microglial activation and was even shown in in vivo studies to reduce glioma proliferation [59,60,61,62,63,64]. This ultimately resulted in three clinical trials (NCT01580969, NCT02272270, and NCT02770378); however, each showed no clear clinical benefit. Another clinical trial considered PLX3397 (Pexidartinib), a potent CSF1R inhibitor that has been shown to deplete microglia populations [65,66]. However, similar to tetracycline, human studies (NCT01349036) showed no clear benefit of PLX3397 in conferring a survival benefit [66]. Currently, there are no treatments on the market for GBM that target macrophage/microglia polarization [67]. Considering the outcomes of the aforementioned studies, although a promising avenue, TAM targeting is yet to translate to meaningful therapeutics for GBM. However, there are many other targets that have been shown in preclinical studies to be promising targets that have not yet made it to clinical trials for GBM. For example, the CCL2/CCR2 pathway has been shown to be critical in the recruitment of TAMs to the GBM TME (Section 3.1.1); however, there are yet to be clinical trials evaluating targeting this pathway specifically in the context of GBM. PF04136309, a small-molecular inhibitor of CCR2, has been shown to be promising in a recent Phase 1b clinical trial looking at its efficacy in the context of pancreatic ductal adenocarcinoma [68,69]. Additionally, C1142, a rat/mouse chimeric monoclonal antibody, has been shown to neutralize CCL2 in a glioma mouse model, significantly reducing TAM numbers and improving survival [70]. Considering these findings, future work targeting the CCL2/CCR2 pathway and exploring implications on tumor growth and ultimately patient survival in the context of clinical trials would greatly move the field forward [69].

Another suitable target is the CD47/SIRPα complex. Specifically, CD47/SIRPα has been explored for its role in helping glioma cells to evade the phagocytic capabilities of microglia. CD47 is a surface protein that has been shown to be upregulated in tumor cells [71] and subsequently binds the receptor SIRPα on phagocytic cells to block the otherwise ensuing phagocytic response. To explore the effects of CD47 targeting, Hutter et al. constructed a GBM xenograft model which was treated with anti-CD47 antibody [72]. They were able to show that blocking CD47 on the surface of tumor cells induced microglial phagocytosis of GBM cells and diminished overall tumor expansion [72,73]. Retrospective clinical studies have highlighted the significance of CD47 in glioma patients, observing that patients with lower overall survival rates expressed higher levels of CD47 and the expression levels inversely correlated with histopathologic grading [74]. With the promising results shown by targeting the CD47/SIRPα pathway, anti-CD47 treatments must be explored in human studies to evaluate therapeutic potential in treating GBM. More specifically, exploring the utility of anti-CD47 treatments in conjunction with other tumor-specific opsonizing antibodies, radiation exposure, and immunosuppressive cell-depleting reagents will allow us to determine its full range of efficacy as an anti-tumor response [72,75].

Another potential target is Toll-like receptors (TLRs). TLRs are widely expressed in neurons, astrocytes, and microglia, and have been explored as a potential therapeutic target for their central role in immunity. Specifically, TLR activation has been shown to either promote or suppress tumor progression. For example, TLR2 is upregulated by glioma cells to promote the degradation of the extracellular matrix, which promotes further tumor growth. However, activation of TLR2 and TLR9 in microglia has been shown to be associated with increased levels of pro-inflammatory signals, phagocytic activity, and suppressed tumor growth [76,77,78]. To better understand the complex dynamics of TLR signaling and responses in the context of glioma progression, Hu et al. identified the glioma-derived endogenous factors that manipulated TLR2 in a mouse brain tumor slice model. Specifically, Hu et al. were able to show that blocking TLR2 significantly diminished glioma growth ex vivo [76]. Humanized anti-TLR2 antibodies have been validated in terms of safety and tolerability in murine models and in clinical trials of those who underwent renal transplantation [79]. However, anti-TLR2 therapies have not yet been explored in clinical trials for GBM, suggesting an unexplored therapeutic target.

**Table 1:** Immunotherapies targeting microglial pathways in GBM. Here, we present a comprehensive summary of the preclinical and clinical studies that have evaluated the efficacy of select TAM-pathway targets.

Therapeutic	Targeted Pathway	Type of Studies	Outcome
Minocycline (Tetracycline analog)	Inhibition of M1 polarization [62,63]	Clinical trials (NCT01580969, NCT02272270, NCT02770378) In vivo GBM rodent model [64]	Clinical trials not promising In vivo studies suggest it can extend median survival when combined with chemotherapeutic agents
PLX3397	CSF1R inhibition and subsequent microglia depletion	Clinical trial (NCT01349036) [66]	Drug well tolerated, but showed no clinical efficacy
PF-04136309	CCR2 inhibition	Clinical trial for treatment of pancreatic cancer (NCT01413022) [69] Clinical trial for treatment of PDAC (NCT02732938) [68]	Pancreatic cancer trial showed increased proportion of patients achieving partial response PDAC trial showed questionable efficacy and safety profile
C1142	Chimeric monoclonal antibody that neutralizes CCL2	In vivo mouse GL261 glioma and human U87 glioma xenograft models [70]	Significantly prolonged survival of glioma-bearing mice
Hu5F9-G4	Antibody that neutralizes CD47	In vivo human xenograft GBM model [72]	Anti-CD47 treatment conferred microglia-mediated survival benefit
CpG-Stat3 siRNA	Inhibit STAT3, which is needed for GSC maintenance and regulates TLR9	In vivo mouse and human glioma models [79]	Reduced GSC numbers and glioma growth
WP1066	Inhibit STAT3	In vitro and in vivo human glioma U87-MG and U373-MG Clinical trials (NCT01904123, NCT04334863) (PMID: 35214076)	Systemic intraperitoneal administration significantly inhibited growth of glioma xenografts Clinical trials are currently recruiting
Peptide R	CXCR4 antagonist	In vivo mouse U87MG glioma xenograft model (PMID: 27015814)	Reduced CXCR4 expression + cell migration in response to CXCL12. Reduced tumor cellularity Promoted M1-type features Disrupted tumor vasculature
Vanucizumab	Bispecific neutralizing antibody targeting angiopoietin-2 + VEGF	In vivo mouse (Gl261) or human (MGG8) GBM xenografts (PMID: 35214076, 27044098)	Prolonged survival in GBM mice and delayed tumor growth

On the opposite end, activating TLR9 via delivery of CpG DNA has been shown to activate the signal transducer and activator of transcription 1 (STAT1) within GBM TAMs. In turn, STAT1-related proteins promote Th1 cell differentiation and the production of Th1-type cytokines such as IL-1β, IFN-γ, iNOS, and TNF-α. Through upregulating levels of pro-inflammatory cytokines and oxidative burst, STAT1 signaling facilitates the repolarization process of M2 macrophages into an M1 state, which has been shown to correlate with decreased tumor growth (Section 3.2) [80]. While findings point to TLR9 as a potential target in glioma immunotherapy, CpG-ODN treatment in a rat glioma model was shown to increase the size of the tumor after intratumoral injection [81]. Future studies can improve our understanding of the dual role of TLR9 and how to preferentially activate downstream anti-tumoral (i.e., ERK, NF-κB, STAT1) rather than pro-tumoral (i.e., JAK2/STAT3, MMP2,9,13) pathways, which can show clinical benefit for patients with GBM.

While exploiting the anti-tumor capabilities of microglia shows promise in the treatment of GBM, another route of treatment proposes to inhibit the activity of TAMs that have already been programmed with pro-tumoral functions. One such approach is inhibiting CSF-1R, a signaling loop that has been implicated in glioma invasion (Section 3.1.2) [82]. In xenograft and transgenic murine models, the inhibition of CSF-1R was shown to halt glioma progression and growth [65,83]. Specifically, mice treated with CSF-1R inhibitor drugs had significantly lower mortality and lower histopathological grade when compared to control [65]. Interestingly, the quantity of TAMs themselves were not impacted by CSF-1R inhibition due to glioma-supplied factors that supported microglia survival [65]. Preclinical studies of CSF-1R inhibition in combination with inhibition of the IGF-1 receptor (IGF-1R) or Phosphatidylinositol 3-kinase (PI3K) pathway significantly prolonged survival in a murine model of recurrent GBMs, suggesting a method of combinatorial therapies that have yet to be tested clinically for patients with GBM [84]. More recently, Fujiwara et al. showed, using the FDA-approved CSF1/CSF1R signaling inhibitor Pexidartinib (PLX3397), that CSF-1R inhibition reduced M2 polarization, suppressed primary tumor growth, and improved metastasis-free survival in models of osteosarcoma, corroborating previous findings [85]. However, long-term treatment with CSF-1R inhibitors resulted in accumulation of IL4 released from other TME cell types which stimulated TAMs to secrete IGF-1 and conversely sustained the survival and growth of glioma cells. Therefore, future studies using PLX3397 combined with immune checkpoint blockade therapy in addition to conventional radio- and chemotherapy may have beneficial effects in glioma patients and have yet to be tested clinically [85].

In addition to exploiting microglial anti-tumor properties and suppressing their pro-tumoral phenotypes, another treatment approach involves repolarizing microglia from the M2 to M1 phenotype. For example, in 2016, Mercurio et al. showed that targeting CXCR4 resulted in inhibition of an M2-associated phenotype and was associated with a reduction in the proliferation and spread of human GBM cells in vitro and in vivo, suggesting the importance of suppressing the M2-phenotype [86,87]. Although there are very few studies exploring the repolarization treatment approach, the few that have been conducted show significant promise. On such study was conducted by Kloepper et al., in which they showed that Vanucizumab, which targets vascular endothelial growth factor (VEGF) and angiopoietin-2, repolarized microglia toward an M1 anti-tumoral phenotype that ultimately improved the survival time of grafted mice and helped delay GBM growth [67,88]. All in all, these are just some of the many promising therapeutic targets in TAM-pathways that should be explored in future clinical trials and evaluated for their ability to potentially confer a survival benefit in GBM patients. We summarize these therapeutic targets comprehensively in Table 1.

## 5. Conclusions and Future Directions

GBM carries significant morbidity and mortality, with 5-year survival less than 7%. What makes this disease particularly challenging is inevitable recurrence post-treatment, further complicated by vast molecular heterogeneity and a largely immunosuppressive TME. Considering these challenges, research efforts have shifted focus from directly targeting GBM cells to modulating the mediators of the immune response, namely TAMs. Recent studies are increasingly showing the critical role TAMs play in the TME in the context of glioma progression. What makes them particularly interesting is their relatively stable molecular profile, lending support to TAM-targeting as a novel GBM immunotherapy. There exist a myriad of TAM-mediated mechanisms that play crucial roles in promoting glioma progression, as reviewed here, which make for suitable targets in the effort to slow and perhaps inhibit GBM tumor progression. The backbone of immunotherapeutic targeting of TAMs is the presumption that there are certain macrophage populations that promote tumor growth and others that inhibit it. In this review, we largely discuss these opposing functions in the context of the M1/M2 dichotomy. As mentioned earlier, this is a simplification of the fact that TAMs likely presume mixed phenotypes in the TME. However, although TAMs may not neatly fall into either M1 or M2 categorizations, this reductionist approach allows us to focus on pro-tumorigenic microglia pathways and anti-tumorigenic microglia pathways and hence direct our attention to suitable targets in future immunotherapies. Here, we reviewed a number of such targets that have been explored in preclinical studies and extracranial tumors and show therapeutic potential in treating GBM. Ultimately, we believe that TAM-targeting holds significant promise in GBM management, and translating aforementioned targets from preclinical studies to clinical trials may offer significant insights into how we may better affect glioma progression and ultimately improve GBM patient outcomes.

## Data Availability

The data are contained within the paper.

## Nomenclature

**Term**	**Abbreviation**
Glioblastoma	GBM
World Health Organization	WHO
Temozolomide	TMZ
Central nervous system	CNS
Central nervous system-associated macrophages	CAMs
Tumor-associated macrophages	TAMs
Glial cell line-derived neurotrophic factor	GDNF
G-protein coupled receptors	GPCRs
Major histocompatibility complex	MHC
Interleukin	IL
Tumor necrosis factor alpha	TNFα
Macrophage inflammatory protein	MIP
Monocyte chemoattractant protein	MCP
Insulin growth factor 1	IGF-1
Alzheimer’s disease	AD
Parkinson’s disease	PD
Regulatory T-cells	Tregs
Myeloid-derived suppressor cells	MDSCs
Matrix metalloprotein	MMP
C-C motif chemokine ligand 2	CCL2
C-C motif chemokine receptor 2	CCR2
C-X3-C motif chemokine ligand 1	CX3CL1
C-X3-C motif chemokine receptor 1	CX3CR1
Colony-stimulating factor 1	CSF1
Macrophage inhibitory cytokine-1	MIC-1
Extracellular vesicles	EV
GBM-derived stem cells	GSC
microRNA	miRNA
T helper 1	Th1
Natural killer	NK
Toll-like receptors	TLRs
Vascular endothelial growth factor	VEGF
